# miR-124 as a Liquid Biopsy Prognostic Biomarker in Small Extracellular Vesicles from NSCLC Patients

**DOI:** 10.3390/ijms241411464

**Published:** 2023-07-14

**Authors:** Darío Sanchez-Cabrero, Álvaro Garcia-Guede, Miranda Burdiel, Olga Pernía, Julián Colmenarejo-Fernandez, Laura Gutierrez, Oliver Higuera, Isabel Esteban Rodriguez, Rocío Rosas-Alonso, Carlos Rodriguez-Antolín, Itsaso Losantos-García, Olga Vera, Javier De Castro-Carpeño, Inmaculada Ibanez de Caceres

**Affiliations:** 1 Biomarkers and Experimental Therapeutics in Cancer, IdiPAZ, 28046 Madrid, Spain; 2 Medical Oncology Department, La Paz University Hospital, 28046 Madrid, Spain; 3 Cancer Epigenetics Laboratory, INGEMM, La Paz University Hospital, 28046 Madrid, Spain; 4 Pathology Department, La Paz University Hospital, 28046 Madrid, Spain; 5 Biostatistics Unit, La Paz University Hospital, 28046 Madrid, Spain

**Keywords:** miR-124, liquid biopsy, NSCLC, sEVs

## Abstract

Despite advances in non-small cell lung cancer (NSCLC) research, this is still the most common cancer type that has been diagnosed up to date. microRNAs have emerged as useful clinical biomarkers in both tissue and liquid biopsy. However, there are no reliable predictive biomarkers for clinical use. We evaluated the preclinical use of seven candidate miRNAs previously identified by our group. We collected a total of 120 prospective samples from 88 NSCLC patients. miRNA levels were analyzed via qRT-PCR from tissue and blood samples. miR-124 gene target prediction was performed using RNA sequencing data from our group and interrogating data from 2952 NSCLC patients from two public databases. We found higher levels of all seven miRNAs in tissue compared to plasma samples, except for miR-124. Our findings indicate that levels of miR-124, both free-circulating and within exosomes, are increased throughout the progression of the disease, suggesting its potential as a marker of disease progression in both advanced and early stages. Our bioinformatics approach identified KPNA4 and SPOCK1 as potential miR-124 targets in NSCLC. miR-124 levels can be used to identify early-stage NSCLC patients at higher risk of relapse.

## 1. Introduction

Lung cancer was the second most frequently diagnosed and the most lethal cancer worldwide in 2020, with an estimated 2.2 million new diagnoses and 1.8 million deaths in recent years [1]. Among lung cancers, non-small cell lung cancer (NSCLC) is the most frequent type, accounting for 84% of all lung cancer diagnoses, with a survival rate at 5 years of only 24%. Late diagnosis and innate or acquired anti-cancer drug resistance are the main causes of the high mortality among men and women. Despite advances in lung cancer therapy, only about 15% of diagnosed patients can benefit from the use of targeted therapies [2]. The remaining NSCLC tumors continue to be treated with platinum derivatives in combination with other drugs [3]. Unfortunately, after continued exposure to the drug, most patients eventually develop resistance, leading to disease progression. Such resistance by tumor cells is usually a multifactorial process involving various adaptive mechanisms, such as the modification of transporter proteins in the membrane or increased DNA repair via the nucleotide excision mechanism or epigenetic changes. Therefore, the identification of reliable diagnostic biomarkers and effective therapeutic strategies is an unmet medical need in lung cancer. In this scenario, epigenetic biomarkers are proposed as an alternative to classical ones [4]. Among the epigenetic changes described in cancer, the role played by microRNAs (miRNAs) has become increasingly relevant [5,6]. miRNAs are non-coding RNAs of 19–23 nucleotides that regulate gene expression via base complementarity, mainly in the 3′ untranslatable region (3′UTR) of target RNA. While the detection of miRNAs in tumor samples has contributed to a better characterization of the oncogenic process, their use as liquid biopsy biomarkers has only started being explored recently [7,8]. This technique avoids tumor heterogeneity, which is very common in solid tumors, provides real-time patient follow-up, and is a much less invasive process than traditional biopsies, with practically zero morbidity. Up to date, the main target of this technique has been circulating tumor DNA (ctDNA), which is released in higher amounts into the bloodstream of cancer patients than healthy ones. However, in recent years, highly sensitive techniques have been developed and the detection of miRNAs in the bloodstream is more readily available [9,10]. Although miRNAs can be free-circulating in the blood, miRNAs are usually exported in small extracellular vesicles (sEVs) or are associated with a ribonucleoprotein complex, argonaute-2, to prevent their degradation [11,12]. The secretion of sEVs, including exosomes, into the blood and other fluids occurs physiologically in the body, but excretion patterns may vary in pathological situations [13]. Indeed, tumor cells release large numbers of sEVs compared to normal cells [14]. The content of these sEVs is enriched in molecules that the tumor cells export to either signal to other regions of the body or to discard tumor-suppressing molecules, including miRNAs [11]. This makes sEVs a good target for the measurement of epigenetic markers via liquid biopsy. However, its use in cancer is not yet widely spread due to the lack of reliable biomarkers for the disease. In this work, we studied the potential use of seven miRNAs previously identified by our group, miR-7, miR-132, miR-335, miR-148, miR-10a, miR-124, and miR-9, as potential novel liquid biopsy biomarkers for the progression of NSCLC [6].

## 2. Results

### 2.1. Descriptive Analysis of Patient Cohorts

**Figure 1:**
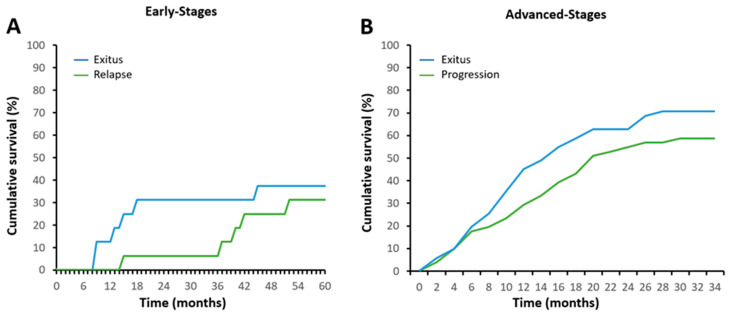
Cumulative survival in terms of relapse and exitus in NSCLC patients with a follow-up of 60 months in early stages and 34 months in advanced stages: (**A**) Early-stage patients. N = 16. (**B**) Advanced-stage patients. N = 51.

This study includes three cohorts of NSCLC patients, two cohorts of early-stage NSCLC patients (I and II), and one cohort of advanced-stage patients (III and IV) (Table 1 and Supplementary Tables S1 and S2). All of the early-stage patients from the exploratory cohort (A, n = 16, Table 1) underwent surgery, achieving complete remission for at least 6 months, and were monitored for 60 months (5 years) via computed tomography or until relapse. By the end of the follow-up, 37.5% of the patients had relapsed with a mean time of 18.7 months, and 31.1% had presented an average time to death of 19.8 months (Figure 1A). The advanced-stage patients (B, n = 51, Table 1) were monitored for 34 months (2.8 years) via computed tomography from the start of the oncological treatment. By the end of the follow-up, 70.6% of patients had relapsed, with an average time of 10.5 months, and 58.8% had died, with a median time of 20 months (Figure 1B). The second early-stage cohort (C, n = 21, Table 1) included prospective patients monitored for at least 15 months. No significant clinical events were observed during that time and, therefore, this cohort was used exclusively for the measurement of miRNA levels in sEVs.

### 2.2. miR-124 Levels Are Associated with Relapse and Exitus in Early-Stage NSCLC Disease

**Figure 2:**
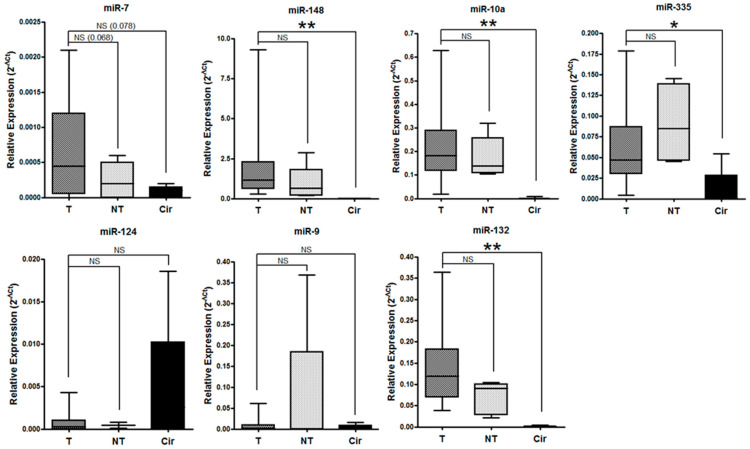
Candidate miRNA levels in tumor tissue (T), adjacent non-tumor tissue (NT), and serum (Cir) samples. The relative levels of miR-7, miR-148, miR-10a, miR-335, miR-124, miR-9, and miR-132 measured via qRT-PCR and calculated using 2^−ΔCt^ are represented. Box-plot graph shows the median with quartile Q1 and Q3. Error bars represent the minimum and maximum values. Mean comparison using Wilcoxon test. *: *p* < 0.05; **: *p* < 0.005; NS: not significant.

We first measured the levels of the seven microRNAs previously identified by our group [6] in cohort A via qRT-PCR. We analyzed their levels in three different conditions for each patient: tumor tissue (T), adjacent non-tumor tissue (NT), and serum (Cir). We observed significantly higher levels in the tissue tumor samples compared to serum for miR-148 (*p* = 0.001), miR-10a (*p* = 0.003), miR-335 (*p* = 0.046), and miR-132 (*p* = 0.002). Conversely, we found a trend toward higher levels of miR-124 in the serum compared to the tumor and non-tumor samples, and for miR-9 in the non-tumor compared to the tumor and the serum samples (Figure 2). No statistical differences were found when comparing normal and tumor tissues. Interestingly, miR-7 showed very low levels in all of the samples when compared with the other microRNAs analyzed. Therefore, we discarded miR-7 for the following analysis.

**Figure 3:**
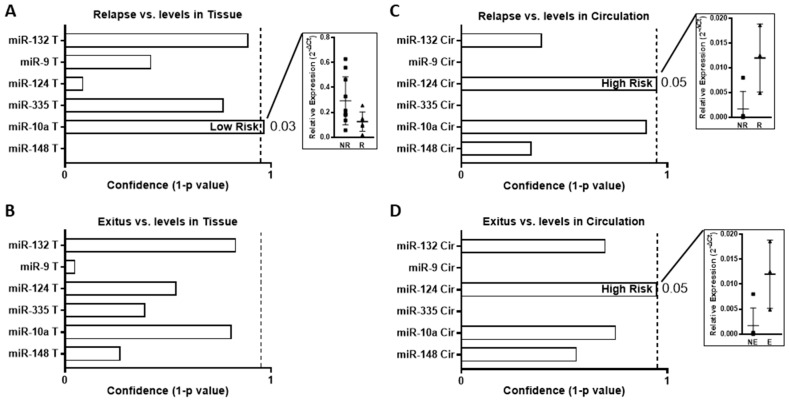
Association between the expression levels of the miRNAs and relapse or exitus in 16 early-stage NSCLC patients. (**A**) Comparison of miRNA levels in tissue and miRNAs with relapse event. (**B**) Comparison of miRNA levels in tissue with exitus event. (**C**) Comparison of miRNA levels in circulation with relapse event. (**D**) Comparison of miRNA levels in circulation with exitus event. Bar-plot graphs show the confidence levels (1-*p*-value) of the mean comparison Mann–Whitney U test between the no-event group vs. the event group: NR: no relapse, R: relapse, NE: no exitus, E: exitus. The *p*-value of significant analyses and the association of miRNA expression with risk are indicated. The dotted line indicates 95% confidence, i.e., *p*-value = 0.05. Dot-plot graphs show the relative expression (mean and SD) measured via qRT-PCR and calculated using 2^−ΔCt^ of significant analyses.

Next, we studied the potential predictive role of the patients’ prognoses of our candidates in cohort A, in terms of relapse events or death (Figure 3). High levels of miR-10a in the tumor samples were associated with a low risk of relapse (*p* = 0.03) (Figure 3A). The levels of miR-132 in the tumors showed a similar trend to miR-10a, with its low levels being associated with a lower number of relapse and death events, but without significant differences (Figure 3A). There were no significant differences between any candidate and death event at the level of tumor tissue (Figure 3B). Conversely, high levels of miR-124 in the serum samples were associated with a high risk of relapse (*p* = 0.05) and exitus (*p* = 0.05) (Figure 3C,D).

We analyzed the correlation of miR-124 levels with all of the other miRNAs to understand potential convergences amongst these miRNAs (Supplementary Figure S1A). We found a significant negative correlation between the levels of free-circulating miR-124 and miR-132 (Corr = −0.73, *p* = 0.03) but no correlation with the other miRNAs analyzed (Supplementary Figure S1A,B). We also observed a significant positive correlation between the levels of miR-124 in the tumor and free-circulating miR-132 (Corr = 0.76, *p* = 0.006) (Supplementary Figure S1B). Interestingly, we found a negative correlation between the tumor and circulating miR-124 expression levels, with this situation being unique with respect to the rest of the analyzed candidates (Supplementary Figure S1A).

Altogether, these results suggest that miR-124 is a microRNA with potential as a liquid biopsy biomarker, since it was the only miRNA with high levels in circulation that were associated with a prognosis of NSCLC.

### 2.3. miR-124 Levels in Liquid Biopsy as a Prognostic Biomarker for PFS and OS in Advanced-Stage NSCLC Disease

**Figure 4:**
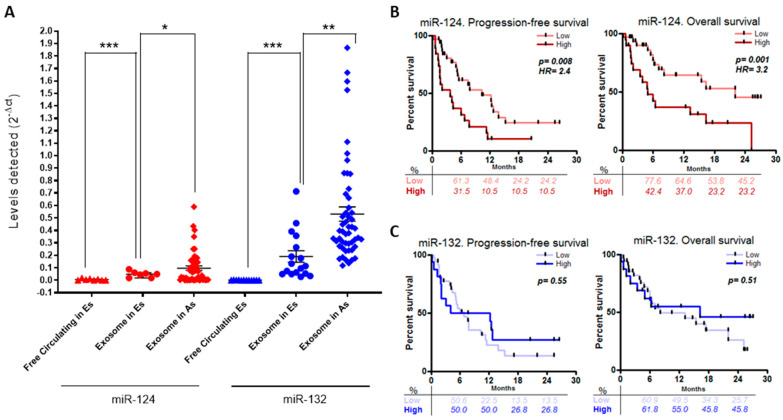
Implications of miR-124- and miR-132-like liquid biopsy markers in advanced-stage NSCLC disease. (**A**) Comparison of relative expression detection levels of miR-124 and miR-132 in free-circulating serum samples from early stages (Es); EV plasma samples of another early-stage cohort and of advanced stages (As). These data were measured via qRT-PCR and calculated using 2-ΔCt. Mean comparison via Student’s t or Mann–Whitney U test. Kaplan–Meier survival analysis in 51 advanced-stage NSCLC samples using the mean of the expression levels of miR-124 (**B**) and miR-132 (**C**) as the cut-off point for the subgroups. Terms of relapse (progression-free survival, PFS) and exitus (overall survival, OS) are represented, and a *p*-value < 0.05 was considered to be significant. *: *p* < 0.05; **: *p* < 0.005; ***: *p* < 0.001. HR: hazard ratio.

Biomarkers within extracellular vesicles have been reported to be more abundant and stable compared to free-circulating biomarkers in the bloodstream [15]. To further validate the role of miR-124 as a biomarker for NSCLC progression, we decided to analyze the levels of miR-124 in sEV content in the alternative early-stage cohort C (Table 1 and Supplementary Table S3). In addition, we included miR-132 given the negative relationship found with miR-124, which suggests a controversial role for miR-132 when compared with the available literature. Figure 4A shows a significantly higher detection of both miR-124 (*p* < 0.001) and miR-132 (*p* < 0.001) in sEV samples compared to free-circulating samples in the early stages. Having determined a higher throughput, we assessed the relative levels of miR-124 and miR-132 in circulating sEVs from cohort B of 51 advanced-stage NSCLC patients (Table 1 and Supplementary Table S2). The levels of both miRNAs were significantly elevated in advanced tumors compared to localized tumors (*p* = 0.017 and *p* = 0.001, respectively) (Figure 4A). Kaplan–Meier survival analyses of these patients show that only miR-124 has significant implications on NSCLC disease (Figure 4B). Patients with lower levels of sEV-miR-124 have a longer time of progression-free survival (PFS) (*p* = 0.008) and overall survival (OS) (*p* = 0.001). In addition, high levels of miR-124 increase the associated risk of relapse by 2.4 times and death by 3.2 times compared with patients with lower levels. We did not find any relationship between miR-132 levels and patient prognosis (Figure 4C).

### 2.4. KPNA4 and SPOCK1 Are Potential miR-124-Regulated NSCLC Oncogenes

**Figure 5:**
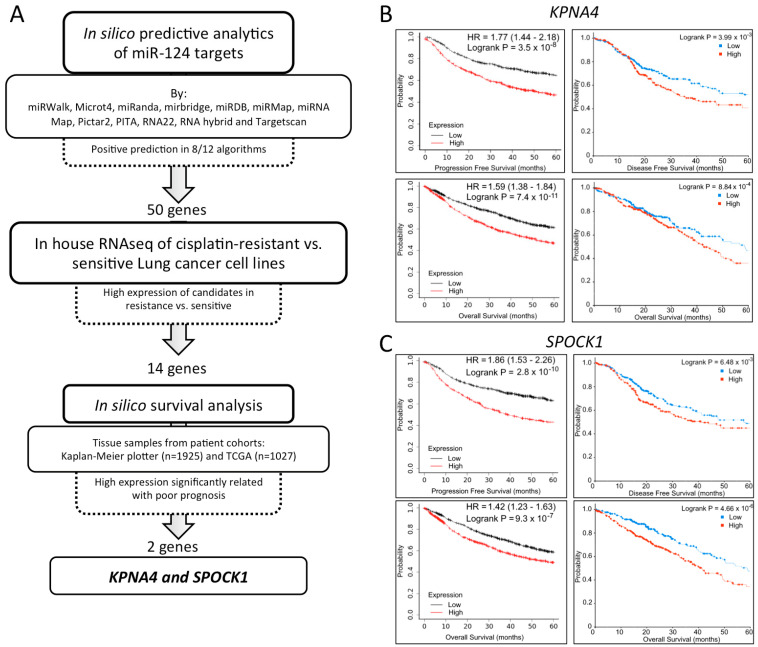
In silico identification of miR-124 target genes with potential implications in NSCLC. (**A**) Bioinformatic analysis workflow to discover KPNA4 and SPOCK1 as potential target genes of miR-124. (**B**,**C**) In silico survival analysis of KPNA4 (**B**) and SPOCK1 (**C**) expression in two cohorts, Kaplan–Meier plotter (left, n = 1925), and TCGA (right, n = 1027) of NSCLC patients in terms of progression-free survival (PFS), disease-free survival (DFS), and overall survival (OS).

Having identified a potential clinical use of miR-124 in NSCLC, we characterized the possible target genes upon which it could be acting. We predicted the binding of miR-124 to the 3′UTR of mRNAs using web-based tools, obtaining a total of 50 candidate genes potentially regulated by miR-124 (Supplementary Table S4). We next compared the expression of these 50 genes in cisplatin-resistant and -sensitive NSCLC cell lines through RNA sequencing. Based on our previous study [6] and the fact that miR-124 is enriched in the sEVs outside of the tumor cell, we focused on genes that had an increased expression in resistant vs. sensitive cells, thus acting as oncogenes. We observed that 11 out of the 50 identified candidates of miR-124 had an increased expression in the NSCLC cisplatin-resistant cell line H23 when compared to the sensitive counterpart. Finally, to confirm their importance in lung cancer progression, we performed Kaplan–Meier survival analysis on tumor tissue samples from a total of 2952 NSCLC patients from public databases according to the expression levels of these 11 genes (Supplementary Table S4). Of them, only *KPNA4* and *SPOCK1* showed a significant relationship between a high gene expression and worse prognosis. Specifically, a high expression of *KPNA4* (Figure 5B) and *SPOCK1* (Figure 5C) was associated with shorter PFS and OS in both in silico cohorts. These results suggest that lower levels of miR-124 induce the expression of *KPNA4* and *SPOCK1* and promote more aggressive behavior of NSCLC.

## 3. Discussion

The search for new biomarkers to predict the behavior of oncologic diseases is a continuously growing area. Current models in lung cancer are mainly based on clinicopathological criteria, but they are still insufficient to assess tumor behavior [16,17]. Recent studies point to epigenetic dysregulation as being one of the main promoters of carcinogenesis. Particularly, the role played by miRNAs has become more relevant in this scenario [5,6], considering their use as tissue and liquid biopsy biomarkers in cancer [8,18]. Liquid biopsy offers a number of advantages compared to direct tissue sampling, namely the avoidance of tumor cell heterogeneity and non-invasive sample acquisition [19]. This work aims to identify clinically relevant liquid biopsy biomarkers in both early and advanced stages of NSCLC based on microRNA levels in blood and sEVs by studying a panel of seven miRNAs involved in cancer progression, previously identified by our group [6].

To identify the best candidates from this panel, it was critical that the exploratory cohort was well standardized. Therefore, we selected patients with localized stages (stages I and II) who underwent surgery. In this group, prognosis was less influenced by biases, depending primarily on the tumor stage and the therapeutic modality [17], in contrast to what occurs in advanced disease. The analysis of both the clinical characteristics of the group and the observed survival were very similar to historical series [20]. We recognize the limitations regarding sample size and types encountered in the pilot study. Due to the scarcity of early-stage NSCLC patients and the need for tissue diagnosis, we made every effort to maximize the sample size of our first cohort within these constraints, pairing fresh frozen tumor and non-tumor tissues, as well as prospective blood serum, which are valuable samples in this stage of the illness. We took steps to address these limitations and provided a comprehensive explanation of our methodology. Despite the constraints, we believe that (1) the selection made allowed for a good level of internal validity by reducing possible clinical–therapeutic confounding variables and (2) the inclusion of these samples offers valuable insights into miRNA levels and their potential implications in lung cancer progression.

miRNAs are well-known epigenetic regulators that can exert their function at the intracellular level. Therefore, we hypothesized that their levels in tumor tissues would be higher than in plasma, supporting their intracellular role. Indeed, we found higher levels in the tumor samples than in plasma for most of our candidates. In this study, miR-7 showed the lowest levels of all of the analyzed candidates, consistent with the regulation of miR-7 via hypermethylation in NSCLC [6], and therefore we discarded it for further analysis. miR-148 was the candidate with the highest expression at the tissue level with significantly low levels in circulation. While miR-148 can be considered to be a tissue-specific tumorigenic microRNA [21], previous studies on NSCLC have indicated the tumor suppressive role of miR-148 in tumor samples [22,23], also showing low detection levels in blood [24]. We observed a trend of increased levels of miR-10a in tumor compared to non-tumor tissue, consistent with previous reports [25]. However, we found an association of high miR-10a levels in tumors with a lower risk of relapse, in contrast with other reports showing that miR-10a is an oncomir associated with increased TNM and lymph node metastasis in NSCLC [25,26]. These controversial results might be due to the different characteristics of the cohorts analyzed: while our exploratory cohort only included early stages, previous reports analyzed early and advanced stages as a whole. Moreover, the fact that we did not find significant associations between the levels of free-circulating miR-10a and clinicopathological parameters in the exploratory cohort reduced the interest of using this miRNA as a liquid biopsy biomarker. Similarly, the circulating levels of miR-148, miR-335, or miR-9 did not show any significant association with the patient’s prognosis, and therefore we discarded the four of them in our study. A table summarizing the role of these miRNAs in NSCLC and the validation in our study is shown in Table 2.

The role of miR-132 in cancer is also controversial [94,95]. In our exploratory cohort, miR-132 showed a trend to lower levels in non-tumor compared to tumor tissues, and there was a significant difference between tumor tissue and free-circulating miR-132, with its levels being lower in plasma. We did not find a significant association between free-circulating miR-132 levels and clinicopathological features. However, the correlation between miR-124 and miR-132 that we observed made us wonder whether these miRNAs could have joint action in NSCLC. Therefore, we also included miR-132 in the next validation steps to evaluate its potential as a biomarker in NSCLC.

Elevated levels of miR-124 in plasma were associated with poor prognosis in early-stage NSCLC in our study. Notably, we observed a negative correlation between free-circulating miR-124 levels and miR-124 levels in tumor tissues. Studies in other tumor types have also observed reduced intracellular levels without a correlation between tissue and plasma levels [96,97]. This disparity between intra- and extracellular levels has been documented for other miRNAs such as miR-125b, miR-143, and miR-221 in prostate cancer [98]. Our results raise the hypothesis that neoplastic cells use different mechanisms to support their proliferation, such as passive release during cell death, release via sEVs, or transport through transmembrane proteins [13]. The decreased levels in the intracellular compartment coinciding with high levels in the bloodstream suggest the secretion of miR-124 through sEVs by the tumor cell, favoring the acquisition of greater aggressiveness by the neoplastic cell.

Moreover, it is known that the levels of free-circulating miRNAs, especially in cancer [14], are much lower than those identified in the sEV context [11]. To optimize detection, we measured miR-124 and miR-132 levels in the sEV content isolated from an alternative early-stage cohort and one advanced-stage cohort. This confirmed greater detection in the sEV content of miR-124 and miR-132 in the early-stage cohort when compared with the free-circulating miRNAs. More importantly, we observed a worse prognosis for patients with elevated sEV miR-124 levels in the advanced-stage cohort. Patients with above-median sEV levels had a poorer prognosis, with a 2.4 times higher risk of progression and 3 times higher mortality than patients with below-median levels, in whom the risk of death was reduced by almost 70%. Furthermore, having shown that the sEV content of these patients is rich in miR-124, the fact that other authors have found an opposite prognostic risk when using the tissue expression levels of miR-124 [99], and that miR-124 can act as a negative regulator of metastasis in lung cancer through targeting the GTPases responsible for exosome export [66,100], supports our hypothesis regarding the active expulsion of tumor suppressor miRNAs by cells during carcinogenesis in lung cancer, even in early stages, to promote the progression of the disease. Conversely, we did not find any association with the prognoses of NSCLC patients when we analyzed the levels of miR-132 in sEVs, suggesting that miR-132 might not be a good candidate for monitoring advanced NSCLC progression. While our data and the literature do not explain the negative correlation observed between miR-124 and miR-132 in terms of circulation, several reports have shown that both miRNAs are potential tumor suppressors that regulate epithelial to mesenchymal transition (EMT) in cancer through the ZEB2/SMAD2/TGF-β pathway [78,101].

To further characterize the role played by miR-124 in NSCLC, we studied the downstream effectors of miR-124 via in silico analysis using miRNA binding prediction tools, in house RNAseq data, and publicly available databases. We found *SPOCK1* and *KPNA4* as potential miR-124-regulated oncogenes in NSCLC. *SPOCK1* (SPARC/osteonectin, cwcv, and kazal-like domain proteoglycan 1) is a matricellular glycoprotein involved in cell proliferation, differentiation, and apoptosis. In NSCLC, *SPOCK1* expression is significantly higher in tumor than in non-tumor tissues [102]. In addition, *SPOCK1* is a novel TGF-β target gene that regulates the EMT of lung cancer cells [103,104]. *KPNA4* (karyopherin subunit alpha 4) is a cytoplasmic protein involved in nuclear import. In NSCLC, its expression is increased and it is epigenetically regulated by several miRNAs to promote proliferation, migration, and tumor growth [105,106]. Further studies will address the possible role of *SPOCK1* and *KPNA4* as new therapeutic targets in NSCLC.

This study is the first to identify miR-124 as a liquid biopsy biomarker of poor prognosis in NSCLC without driver mutations, regardless of stage. While the potential role of miR-124 as a tumor suppressor miRNA in cancer has been previously shown in various tumor types such as lung, colorectal cancer, brain tumors, osteosarcoma, and breast cancer [107,108,109,110,111,112], none of them have established a correlation between elevated levels of this miRNA in circulation, free, or exosomal forms and worse prognosis in these pathologies. Our study highlights the potential specificity of miR-124 to lung cancer based on the current results and our previous screening, in which miR-124 showed altered levels specifically in lung cancer cell lines [6]. In this work, we focused on the potential use of miR-124 as a non-invasive biomarker of disease progression in non-small cell lung cancer patients. It is of critical importance to identify markers that can predict patient outcomes from the early stages, as currently there are no reliable means to identify patients at higher risk of recurrence or determine the most appropriate therapeutic regimen for early-stage patients after surgery. The measurement of miR-124 in sEVs would help to identify patients with more aggressive tumors, as well as to monitor their response to treatment, both in localized and metastatic stages. To date, little is known about the specific role of exporting miRNAs in sEVs. However, understanding their mechanism of action and the consequences derived from their dysregulation will provide a complete vision of the intricate machinery of carcinogenesis and will help to design new methods of detection, monitoring, and even treatment.

## 4. Materials and Methods

### 4.1. Sample Collection and Ethical Aspects

Tissue and plasma samples were obtained from 88 patients diagnosed with NSCLC without harboring driver mutations, from the Thoracic Surgery and the Medical Oncology Services of the University Hospital La Paz, and the patients were subdivided into early-stage (one exploratory cohort of 16 patients and one validation cohort of 21 patients) and advanced-stage patients (one validation cohort of 51 patients). All of the samples were processed following the standard operating procedures with the appropriate approval of the human research ethics committees, including informed consent within the context of research (HULP: PI-2109). Additional details are fully described in the Supplementary Materials and Methods.

### 4.2. miRNA Extraction

At the time of collection, the tissue was immediately frozen and stored at −80 °C. Total RNA, including miRNAs, from tumor and non-tumor tissue was isolated and purified using the miRNeasy Mini Kit (Qiagen, Hilden, Germany) combined with DNase treatment to ensure that only RNA molecules were present. Blood samples were processed within the first 30 min after collection using Vacutainer EDTA blood collection tubes. Unbroken cells and debris were removed from the blood plasma samples via two centrifugations and then stored at −80 °C until use. Free-circulating miRNAs in serum were isolated from 1 mL of sample using the QIAamp Circulating Nucleic Acid protocol. sEVs-miRNAs from blood plasma were extracted using the exoRNeasy Serum/Plasma Midi Kit (Qiagen, Hilden, Germany), including a 0.22 µm filtration step, following product directions. In all cases, RNA concentration and integrity were quantified using NanoDrop ND-1000 (Thermo Fisher Scientific, Waltham, MA, USA). Additional information is fully described in the Supplementary Materials and Methods.

### 4.3. qRT-PCR

The non-specific retrotranscription and quantitative PCR were conducted using the TaqMan^TM^ Advanced miRNA cDNA Synthesis Kit and Advanced miRNA assay for each candidate (Thermo Fisher Scientific, Waltham, MA, USA). Samples were analyzed in triplicate using the HT7900 Real-Time PCR system (Applied Biosystems, Waltham, MA, USA), and relative expression levels were calculated according to the comparative threshold cycle method 2^(−ΔCt)^ using miR-25 or an experimentally identified sEVs-miRNA as a reference miRNA. Additional details are fully described in the Supplementary Materials and Methods.

### 4.4. Identification of microRNA Target Genes

Bioinformatic predictive analytics of miR-124 gene targets. Predictions of the interaction algorithms for miRNAs and their 3′UTR regions were made by interrogating miRWalk v2. Genes for which the binding prediction was positive in at least 8 of these 12 algorithms were then selected.

RNA Sequencing. RNA extraction from cisplatin-resistant and cisplatin-sensitive human NSCLC cell lines, assessment of quality, library preparation, normalization, and analysis of RNAseq are described in the Supplementary Materials and Methods.

In silico databases. We obtained survival data based on the mean of the expression data for each gene using the KMplot and cBioportal tools. A *p*-value (log rank test) < 0.05 was considered to be significant in all of the survival analyses. Additional information is fully described in the Supplementary Materials and Methods.

### 4.5. Statistical Analysis

Qualitative data are described as absolute frequencies and percentages and quantitative data are described as mean ± standard deviation or median and quartiles. The association between qualitative variables was analyzed using the chi-square test or Fisher’s exact test. For comparisons between continuous variables, the Spearman correlation test was used. Comparisons between categorical and continuous variables were made using the Mann–Whitney U test and Wilcoxon signed-rank test. Survival analysis was performed using Kaplan–Meier analysis, comparing survival functions by group using log rank tests. We used the mean expression as a cut-off point to subdivide the groups between high and low miR-124 or miR-132 levels. The risk associated with the variables of interest was analyzed using Cox regression. All of the statistical tests were considered to be bilateral and *p*-values of less than 0.05 were considered to be significant. The data were analyzed using SAS 9.3 statistical software (SAS Institute, Carly, NC, USA).

## Figures and Tables

**Table 1:** Demographics and clinicopathological data of exploratory cohort (A) and validation cohort (B, C) NSCLC patients. COPD, chronic obstructive pulmonary disease; NS, not specified; ND, not determined.

Early-Stage Exploratory Cohort (A, n = 16)		Advanced-Stage Cohort (B, n = 51)		Early-Stage Cohort (C, n = 21)	
**Age** (median)	62	**Age** (median)	65	**Age** (median)	67
**Sex**		**Sex**		**Sex**	
Male	12 (75%)	Male	34 (66%)	Male	10 (48%)
Female	4 (25%)	Female	17 (34%)	Female	11 (52%)
**Smoking**		**Histology**		**Smoking**	
Active	5 (31%)	Adeno	30 (59%)	Active	9 (43%)
Ex-smoker	11 (69%)	Squamous	14 (27%)	Ex-smoker	7 (33%)
**COPD**		NS	7 (14%)	Other	5 (24%)
Yes	6 (37%)	**Stages**		**COPD**	
No	10 (36%)	III	22 (4%)	Yes	7 (33%)
**Histology**		IV	26 (51%)	No	14 (67%)
Adeno	12 (75%)	ND	3 (6%)	**Histology**	
Squamous	3 (19%)			Adeno	17 (81%)
NS	1 (6%)			Squamous	4 (19%)
**Stages**				**Stages**	
Ia	8 (50%)			Ia	10 (48%)
Ib	3 (19%)			Ib	4 (19%)
IIa	1 (6%)			IIa	2 (9%)
IIb	4 (25%)			IIb	5 (24%)
**LV invasion**				**Adyuvant Qtx**	
Yes	6 (37%)			Yes	8 (38%)
No	10 (63%)			No	13 (62%)
**Adyuvant Qtx**					
Yes	6 (37%)				
No	10 (63%)				

**Table 2:** Summary of the roles of the seven miRNAs analyzed in NSCLC.

miRNA	Tumor Suppressive Effect	References	Validated in Our Study for Liquid Biopsy
miR-7	Tumorigenesis, progression, growth, therapy resistance, sensitivity, metastasis, metabolism, and proliferation.	[27,28,29,30,31,32,33,34,35,36,37,38,39,40,41,42,43,44,45,46,47,48,49,50,51,52]	Levels too low
miR-9	Controversial roles; it acts as a tumor suppressor and oncogene depending on the context.	[53,54,55,56,57,58,59,60,61]	No association
miR-10	Proliferation, metastasis, and therapy sensitivity.	[26,62,63,64,65]	No association
miR-124	Metastasis, therapy sensitivity, progression, invasion, cancer development, tumorigenesis, proliferation, migration, and EMT.	[64,66,67,68,69,70,71,72,73,74]	Predictive of NSCLC prognosis
miR-132	Proliferation, EMT, migration, and invasion.	[75,76,77,78,79,80,81,82]	No association
miR-148	Proliferation.	[83,84]	No association
miR-335	Therapy sensitivity, progression, EMT, development, proliferation, apoptosis, and invasion.	[85,86,87,88,89,90,91,92,93]	No association

## Data Availability

The datasets generated and/or analyzed during the current study are available in the Supplementary Files.

## Supplementary Materials

The following supporting information can be downloaded at https://www.mdpi.com/article/10.3390/ijms241411464/s1. References [113,114,115,116,117,118] are cited in the supplementary materials.

## References

[1] Sung H., Ferlay J., Siegel R.L., Laversanne M., Soerjomataram I., Jemal A., Bray F. (2021). Global Cancer Statistics 2020: GLOBOCAN Estimates of Incidence and Mortality Worldwide for 36 Cancers in 185 Countries. CA A Cancer J. Clin..

[2] Soria J.C., Ohe Y., Vansteenkiste J., Reungwetwattana T., Chewaskulyong B., Lee K.H., Dechaphunkul A., Imamura F., Nogami N., Kurata T. (2018). Osimertinib in Untreated EGFR-Mutated Advanced Non-Small-Cell Lung Cancer. N. Engl. J. Med..

[3] Gandhi L., Rodriguez-Abreu D., Gadgeel S., Esteban E., Felip E., De Angelis F., Domine M., Clingan P., Hochmair M.J., Powell S.F. (2018). Pembrolizumab plus Chemotherapy in Metastatic Non-Small-Cell Lung Cancer. N. Engl. J. Med..

[4] Cortes-Sempere M., de Miguel M.P., Pernia O., Rodriguez C., de Castro Carpeno J., Nistal M., Conde E., Lopez-Rios F., Belda-Iniesta C., Perona R. (2013). IGFBP-3 methylation-derived deficiency mediates the resistance to cisplatin through the activation of the IGFIR/Akt pathway in non-small cell lung cancer. Oncogene.

[5] Calin G.A., Dumitru C.D., Shimizu M., Bichi R., Zupo S., Noch E., Aldler H., Rattan S., Keating M., Rai K. (2002). Frequent deletions and down-regulation of micro- RNA genes miR15 and miR16 at 13q14 in chronic lymphocytic leukemia. Proc. Natl. Acad. Sci. USA.

[6] Vera O., Jimenez J., Pernia O., Rodriguez-Antolin C., Rodriguez C., Sanchez Cabo F., Soto J., Rosas R., Lopez-Magallon S., Esteban Rodriguez I. (2017). DNA Methylation of miR-7 is a Mechanism Involved in Platinum Response through MAFG Overexpression in Cancer Cells. Theranostics.

[7] Kodahl A.R., Lyng M.B., Binder H., Cold S., Gravgaard K., Knoop A.S., Ditzel H.J. (2014). Novel circulating microRNA signature as a potential non-invasive multi-marker test in ER-positive early-stage breast cancer: A case control study. Mol. Oncol..

[8] Zhang H., Mao F., Shen T., Luo Q., Ding Z., Qian L., Huang J. (2017). Plasma miR-145, miR-20a, miR-21 and miR-223 as novel biomarkers for screening early-stage non-small cell lung cancer. Oncol. Lett..

[9] Zugazagoitia J., Ramos I., Trigo J.M., Palka M., Gomez-Rueda A., Jantus-Lewintre E., Camps C., Isla D., Iranzo P., Ponce-Aix S. (2019). Clinical utility of plasma-based digital next-generation sequencing in patients with advance-stage lung adenocarcinomas with insufficient tumor samples for tissue genotyping. Ann. Oncol..

[10] Weber J.A., Baxter D.H., Zhang S., Huang D.Y., Huang K.H., Lee M.J., Galas D.J., Wang K. (2010). The microRNA spectrum in 12 body fluids. Clin. Chem..

[11] Pegtel D.M., Cosmopoulos K., Thorley-Lawson D.A., van Eijndhoven M.A., Hopmans E.S., Lindenberg J.L., de Gruijl T.D., Wurdinger T., Middeldorp J.M. (2010). Functional delivery of viral miRNAs via exosomes. Proc. Natl. Acad. Sci. USA.

[12] Valadi H., Ekstrom K., Bossios A., Sjostrand M., Lee J.J., Lotvall J.O. (2007). Exosome-mediated transfer of mRNAs and microRNAs is a novel mechanism of genetic exchange between cells. Nat. Cell Biol..

[13] Huan J., Hornick N.I., Shurtleff M.J., Skinner A.M., Goloviznina N.A., Roberts C.T., Kurre P. (2013). RNA trafficking by acute myelogenous leukemia exosomes. Cancer Res..

[14] Szczepanski M.J., Szajnik M., Welsh A., Whiteside T.L., Boyiadzis M. (2011). Blast-derived microvesicles in sera from patients with acute myeloid leukemia suppress natural killer cell function via membrane-associated transforming growth factor-beta1. Haematologica.

[15] Endzelins E., Berger A., Melne V., Bajo-Santos C., Sobolevska K., Abols A., Rodriguez M., Santare D., Rudnickiha A., Lietuvietis V. (2017). Detection of circulating miRNAs: Comparative analysis of extracellular vesicle-incorporated miRNAs and cell-free miRNAs in whole plasma of prostate cancer patients. BMC Cancer.

[16] Goldstraw P., Crowley J., Chansky K., Giroux D.J., Groome P.A., Rami-Porta R., Postmus P.E., Rusch V., Sobin L., International Association for the Study of Lung Cancer International Staging Committee and Participating Institutions (2007). The IASLC Lung Cancer Staging Project: Proposals for the revision of the TNM stage groupings in the forthcoming (seventh) edition of the TNM Classification of malignant tumours. J. Thorac. Oncol. Off. Publ. Int. Assoc. Study Lung Cancer.

[17] Kelsey C.R., Marks L.B., Glatstein E. (2009). Elective nodal irradiation for locally advanced non-small-cell lung cancer: It’s called cancer for a reason. Int. J. Radiat. Oncol. Biol. Phys..

[18] Okugawa Y., Grady W.M., Goel A. (2015). Epigenetic Alterations in Colorectal Cancer: Emerging Biomarkers. Gastroenterology.

[19] Sempere L.F. (2011). Integrating contextual miRNA and protein signatures for diagnostic and treatment decisions in cancer. Expert. Rev. Mol. Diagn..

[20] Herbst R.S., Heymach J.V., Lippman S.M. (2008). Lung cancer. N. Engl. J. Med..

[21] Chen Y., Song Y.X., Wang Z.N. (2013). The microRNA-148/152 family: Multi-faceted players. Mol. Cancer.

[22] Ge H., Li B., Hu W.X., Li R.J., Jin H., Gao M.M., Ding C.M. (2015). MicroRNA-148b is down-regulated in non-small cell lung cancer and associated with poor survival. Int. J. Clin. Exp. Pathol..

[23] Wang R., Ye F., Zhen Q., Song T., Tan G., Chu W., Zhang Y., Lv B., Zhao X., Liu J. (2016). MicroRNA-148b is a potential prognostic biomarker and predictor of response to radiotherapy in non-small-cell lung cancer. J. Physiol. Biochem..

[24] Huang M.X. (2016). Down-expression of circulating micro ribonucleic acid (miRNA)-148/152 family in plasma samples of non-small cell lung cancer patients. J. Cancer Res. Ther..

[25] Bao M., Pan S., Yang W., Chen S., Shan Y., Shi H. (2018). Serum miR-10a-5p and miR-196a-5p as non-invasive biomarkers in non-small cell lung cancer. Int. J. Clin. Exp. Pathol..

[26] Yu T., Liu L., Li J., Yan M., Lin H., Liu Y., Chu D., Tu H., Gu A., Yao M. (2015). MiRNA-10a is upregulated in NSCLC and may promote cancer by targeting PTEN. Oncotarget.

[27] Zhao J.G., Men W.F., Tang J. (2015). MicroRNA-7 enhances cytotoxicity induced by gefitinib in non-small cell lung cancer via inhibiting the EGFR and IGF1R signalling pathways. Contemp. Oncol..

[28] Zhang X., Yang D., Wei Y. (2018). Overexpressed CDR1as functions as an oncogene to promote the tumor progression via miR-7 in non-small-cell lung cancer. OncoTargets Ther..

[29] Zeng J., Cai S. (2017). Breviscapine suppresses the growth of non-small cell lung cancer by enhancing microRNA-7 expression. J. Biosci..

[30] Xiong S., Zheng Y., Jiang P., Liu R., Liu X., Qian J., Gu J., Chang L., Ge D., Chu Y. (2014). PA28gamma emerges as a novel functional target of tumour suppressor microRNA-7 in non-small-cell lung cancer. Br. J. Cancer.

[31] Xiong S., Zheng Y., Jiang P., Liu R., Liu X., Chu Y. (2011). MicroRNA-7 inhibits the growth of human non-small cell lung cancer A549 cells through targeting BCL-2. Int. J. Biol. Sci..

[32] Su C., Han Y., Zhang H., Li Y., Yi L., Wang X., Zhou S., Yu D., Song X., Xiao N. (2018). CiRS-7 targeting miR-7 modulates the progression of non-small cell lung cancer in a manner dependent on NF-κB signalling. J. Cell. Mol. Med..

[33] Liu R., Liu X., Zheng Y., Gu J., Xiong S., Jiang P., Jiang X., Huang E., Yang Y., Ge D. (2014). MicroRNA-7 sensitizes non-small cell lung cancer cells to paclitaxel. Oncol. Lett..

[34] He X., Li C., Wu X., Yang G. (2015). Docetaxel inhibits the proliferation of non-small-cell lung cancer cells via upregulation of microRNA-7 expression. Int. J. Clin. Exp. Pathol..

[35] Giles K.M., Barker A., Zhang P.M., Epis M.R., Leedman P.J. (2011). MicroRNA regulation of growth factor receptor signaling in human cancer cells. Methods Mol. Biol..

[36] Chou Y.T., Lin H.H., Lien Y.C., Wang Y.H., Hong C.F., Kao Y.R., Lin S.C., Chang Y.C., Lin S.Y., Chen S.J. (2010). EGFR promotes lung tumorigenesis by activating miR-7 through a Ras/ERK/Myc pathway that targets the Ets2 transcriptional repressor ERF. Cancer Res..

[37] Cao Q., Mao Z.D., Shi Y.J., Chen Y., Sun Y., Zhang Q., Song L., Peng L.P. (2016). MicroRNA-7 inhibits cell proliferation, migration and invasion in human non-small cell lung cancer cells by targeting FAK through ERK/MAPK signaling pathway. Oncotarget.

[38] Zhang Y., Qi W., Wu Y. (2023). EIF4A3-induced circular RNA SCAP facilitates tumorigenesis and progression of non-small-cell lung cancer via miR-7/SMAD2 signaling. Environ. Sci. Pollut. Res. Int..

[39] Xiao H. (2019). MiR-7-5p suppresses tumor metastasis of non-small cell lung cancer by targeting NOVA2. Cell. Mol. Biol. Lett..

[40] Woo S.Y., Lee S.Y., Yu S.L., Park S.J., Kang D., Kim J.S., Jeong I.B., Kwon S.J., Hwang W.J., Park C.R. (2020). MicroRNA-7-5p’s role in the O-GlcNAcylation and cancer metabolism. Non-Coding RNA Res..

[41] Su T., Huang S., Zhang Y., Guo Y., Zhang S., Guan J., Meng M., Liu L., Wang C., Yu D. (2022). miR-7/TGF-β2 axis sustains acidic tumor microenvironment-induced lung cancer metastasis. Acta Pharm. Sinica. B.

[42] Rodríguez-Antolín C., Felguera-Selas L., Pernía O., Vera O., Esteban I., Losantos García I., de Castro J., Rosas-Alonso R., Ibanez de Caceres I. (2019). miR-7 methylation as a biomarker to predict poor survival in early-stage non-small cell lung cancer patients. Cell Biosci..

[43] Ma P., Han W., Meng C., Tan X., Liu P., Dong L. (2022). LINC02389/miR-7-5p Regulated Cisplatin Resistance of Non-Small-Cell Lung Cancer via Promoting Oxidative Stress. Anal. Cell. Pathol..

[44] Liu S., Wang W., Ning Y., Zheng H., Zhan Y., Wang H., Yang Y., Luo J., Wen Q., Zang H. (2022). Exosome-mediated miR-7-5p delivery enhances the anticancer effect of Everolimus via blocking MNK/eIF4E axis in non-small cell lung cancer. Cell Death Dis..

[45] Liu B., Li H., Liu X., Li F., Chen W., Kuang Y., Zhao X., Li L., Yu B., Jin X. (2021). CircZNF208 enhances the sensitivity to X-rays instead of carbon-ions through the miR-7-5p/SNCA signal axis in non-small-cell lung cancer cells. Cell. Signal..

[46] Li Q., Wu X., Guo L., Shi J., Li J. (2019). MicroRNA-7-5p induces cell growth inhibition, cell cycle arrest and apoptosis by targeting PAK2 in non-small cell lung cancer. FEBS Open Bio.

[47] Li D., Fu Z., Dong C., Song Y. (2022). Downregulation of circATXN7 represses non-small cell lung cancer growth by releasing miR-7-5p. Thorac. Cancer.

[48] Ku G.W., Kang Y., Yu S.L., Park J., Park S., Jeong I.B., Kang M.W., Son J.W., Kang J. (2021). LncRNA LINC00240 suppresses invasion and migration in non-small cell lung cancer by sponging miR-7-5p. BMC Cancer.

[49] Guo G., Li L., Song G., Wang J., Yan Y., Zhao Y. (2020). miR-7/SP1/TP53BP1 axis may play a pivotal role in NSCLC radiosensitivity. Oncol. Rep..

[50] Guan S., Li L., Chen W.S., Jiang W.Y., Ding Y., Zhao L.L., Shi Y.F., Wang J., Gui Q., Xu C.C. (2021). Circular RNA WHSC1 exerts oncogenic properties by regulating miR-7/TAB2 in lung cancer. J. Cell. Mol. Med..

[51] Chen S., Guan L., Zhao X., Yang J., Chen L., Guo M., Zhao J., Chen C., Zhou Y., Han Y. (2022). Optimized thyroid transcription factor-1 core promoter-driven microRNA-7 expression effectively inhibits the growth of human non-small-cell lung cancer cells. J. Zhejiang University. Sci. B.

[52] Chen R., Qian Z., Xu X., Zhang C., Niu Y., Wang Z., Sun J., Zhang X., Yu Y. (2021). Exosomes-transmitted miR-7 reverses gefitinib resistance by targeting YAP in non-small-cell lung cancer. Pharmacol. Res..

[53] Xu G., Shao G., Pan Q., Sun L., Zheng D., Li M., Li N., Shi H., Ni Y. (2017). MicroRNA-9 regulates non-small cell lung cancer cell invasion and migration by targeting eukaryotic translation initiation factor 5A2. Am. J. Transl. Res..

[54] Xiong K., Shao L.H., Zhang H.Q., Jin L., Wei W., Dong Z., Zhu Y.Q., Wu N., Jin S.Z., Xue L.X. (2018). MicroRNA-9 functions as a tumor suppressor and enhances radio-sensitivity in radio-resistant A549 cells by targeting neuropilin 1. Oncol. Lett..

[55] Wei W., Dong Z., Gao H., Zhang Y.Y., Shao L.H., Jin L.L., Lv Y.H., Zhao G., Shen Y.N., Jin S.Z. (2019). MicroRNA-9 enhanced radiosensitivity and its mechanism of DNA methylation in non-small cell lung cancer. Gene.

[56] Wang H., Wu Q., Zhang Y., Zhang H.N., Wang Y.B., Wang W. (2017). TGF-β1-induced epithelial-mesenchymal transition in lung cancer cells involves upregulation of miR-9 and downregulation of its target, E-cadherin. Cell. Mol. Biol. Lett..

[57] Pan Q., Sun L., Zheng D., Li N., Shi H., Song J., Shao G., Xu G. (2018). MicroRNA-9 Enhanced Cisplatin Sensitivity in Nonsmall Cell Lung Cancer Cells by Regulating Eukaryotic Translation Initiation Factor 5A2. BioMed Res. Int..

[58] Li G., Wu F., Yang H., Deng X., Yuan Y. (2017). MiR-9-5p promotes cell growth and metastasis in non-small cell lung cancer through the repression of TGFBR2. Biomed Pharmacother..

[59] Jin Y., Kang Y., Peng X., Yang L., Li Q., Mei Q., Chen X., Hu G., Tang Y., Yuan X. (2021). Irradiation-Induced Activated Microglia Affect Brain Metastatic Colonization of NSCLC Cells via miR-9/CDH1 Axis. OncoTargets Ther..

[60] Han L., Wang W., Ding W., Zhang L. (2017). MiR-9 is involved in TGF-β1-induced lung cancer cell invasion and adhesion by targeting SOX7. J. Cell. Mol. Med..

[61] Chen X., Zhu L., Ma Z., Sun G., Luo X., Li M., Zhai S., Li P., Wang X. (2015). Oncogenic miR-9 is a target of erlotinib in NSCLCs. Sci. Rep..

[62] Zhu Y., Ma K., Ye Y., Tang J., Zhu J. (2022). Long non-coding RNA LINRIS is upregulated in non-small cell lung cancer and its silencing inhibits cell proliferation by suppressing microRNA-10a maturation. Bioengineered.

[63] Zhang H., Lu Y., Chen E., Li X., Lv B., Vikis H.G., Liu P. (2017). XRN2 promotes EMT and metastasis through regulating maturation of miR-10a. Oncogene.

[64] Lu X., Xue B., Zhang T., Zhou X., Zhang Y. (2019). Down-regulation of microRNA-10a mediates the anti-tumor effect of icaritin in A549 cells via the PTEN/AKT and ERK pathway. Gen. Physiol. Biophys..

[65] Gao Y., Zhao H., Mu L. (2021). LncRNA-KAT7 Negatively Regulates miR-10a Through an Epigenetic Pathway to Participate in Nonsmall Cell Lung Cancer. Cancer Biother. Radiopharm..

[66] Zhu Q., Zhang Y., Li M., Zhang Y., Zhang H., Chen J., Liu Z., Yuan P., Yang Z., Wang X. (2023). MiR-124-3p impedes the metastasis of non-small cell lung cancer via extracellular exosome transport and intracellular PI3K/AKT signaling. Biomark. Res..

[67] Wu J., Weng Y., He F., Liang D., Cai L. (2018). LncRNA MALAT-1 competitively regulates miR-124 to promote EMT and development of non-small-cell lung cancer. Anti-Cancer Drugs.

[68] Tang L.X., Chen G.H., Li H., He P., Zhang Y., Xu X.W. (2018). Long non-coding RNA OGFRP1 regulates LYPD3 expression by sponging miR-124-3p and promotes non-small cell lung cancer progression. Biochem. Biophys. Res. Commun..

[69] Tan X., Zhang C., Gao W., Sun B., Jiang B., Song P. (2021). Overexpression of microRNA-124-5p sensitizes non-small cell lung cancer cells to treatment with 5-fluorouracil via AEG-1 regulation. Oncol. Lett..

[70] Song X., Kong F., Zong Z., Ren M., Meng Q., Li Y., Sun Z. (2019). miR-124 and miR-142 enhance cisplatin sensitivity of non-small cell lung cancer cells through repressing autophagy via directly targeting SIRT1. RSC Adv..

[71] Qi M.M., Ge F., Chen X.J., Tang C., Ma J. (2019). MiR-124 changes the sensitivity of lung cancer cells to cisplatin through targeting STAT3. Eur. Rev. Med. Pharmacol. Sci..

[72] Liu T., Zhu J., Du W., Ning W., Zhang Y., Zeng Y., Liu Z., Huang J.A. (2020). AKT2 drives cancer progression and is negatively modulated by miR-124 in human lung adenocarcinoma. Respir. Res..

[73] Li H., Guo X., Li Q., Ran P., Xiang X., Yuan Y., Dong T., Zhu B., Wang L., Li F. (2018). Long non-coding RNA 1308 promotes cell invasion by regulating the miR-124/ADAM 15 axis in non-small-cell lung cancer cells. Cancer Manag. Res..

[74] Jin H., Li Q., Cao F., Wang S.N., Wang R.T., Wang Y., Tan Q.Y., Li C.R., Zou H., Wang D. (2017). miR-124 Inhibits Lung Tumorigenesis Induced by K-ras Mutation and NNK. Mol. Ther. Nucleic Acids.

[75] Zhang K., Li Y., Qu L., Ma X., Zhao H., Tang Y. (2018). Long noncoding RNA Sox2 overlapping transcript (SOX2OT) promotes non-small-cell lung cancer migration and invasion via sponging microRNA 132 (miR-132). OncoTargets Ther..

[76] Zhang J.X., Zhai J.F., Yang X.T., Wang J. (2016). MicroRNA-132 inhibits migration, invasion and epithelial-mesenchymal transition by regulating TGFβ1/Smad2 in human non-small cell lung cancer. Eur. Rev. Med. Pharmacol. Sci..

[77] Zhang B., Lu L., Zhang X., Ye W., Wu J., Xi Q., Zhang X. (2014). Hsa-miR-132 regulates apoptosis in non-small cell lung cancer independent of acetylcholinesterase. J. Mol. Neurosci. MN.

[78] You J., Li Y., Fang N., Liu B., Zu L., Chang R., Li X., Zhou Q. (2014). MiR-132 suppresses the migration and invasion of lung cancer cells via targeting the EMT regulator ZEB2. PLoS ONE.

[79] Wang N., Xu Y., Guo Q., Zhu C., Zhao W., Qian W., Zheng M. (2021). Effects of miR-132-3p on progress and epithelial mesenchymal transition of non-small cell lung cancer via regulating KLF7. J. Thorac. Dis..

[80] Liu B., Qiang L., Guan B., Ji Z. (2022). Targeting kinesin family member 21B by miR-132-3p represses cell proliferation, migration and invasion in gastric cancer. Bioengineered.

[81] Li Y., Zu L., Wang Y., Wang M., Chen P., Zhou Q. (2015). miR-132 inhibits lung cancer cell migration and invasion by targeting SOX4. J. Thorac. Dis..

[82] Guo H., Zhang X., Chen Q., Bao Y., Dong C., Wang X. (2018). miR-132 suppresses the migration and invasion of lung cancer cells by blocking USP9X-induced epithelial-mesenchymal transition. Am. J. Transl. Res..

[83] Xi Y., Shen W., Jin C., Wang L., Yu B. (2020). PVT1 Promotes the Proliferation and Migration of Non-Small Cell Lung Cancer via Regulating miR-148/RAB34 Signal Axis. OncoTargets Ther..

[84] Lee B.B., Kim D., Kim Y., Han J., Shim Y.M., Kim D.H. (2023). Metformin regulates expression of DNA methyltransferases through the miR-148/-152 family in non-small lung cancer cells. Clin. Epigenetics.

[85] Zhao W., Chen T., Zhao Y. (2020). Upregulated lncRNA CASC9 Contributes to Progression of Non-Small Cell Lung Cancer Through Inhibition of miR-335-3p and Activation S100A14 Expression. OncoTargets Ther..

[86] Yu C., Ying J., Yu K., Shen W., Jiang M. (2022). Circ_0074027 Contributes to Non-Small Cell Lung Cancer Progression by Upregulating CUL4B Expression Through miR-335-5p. Cancer Biother. Radiopharm..

[87] Wang H., Li M., Zhang R., Wang Y., Zang W., Ma Y., Zhao G., Zhang G. (2013). Effect of miR-335 upregulation on the apoptosis and invasion of lung cancer cell A549 and H1299. Tumour. Biol..

[88] Tang H., Zhu J., Du W., Liu S., Zeng Y., Ding Z., Zhang Y., Wang X., Liu Z., Huang J. (2018). CPNE1 is a target of miR-335-5p and plays an important role in the pathogenesis of non-small cell lung cancer. J. Exp. Clin. Cancer Res..

[89] Safaric Tepes P., Pal D., Lindsted T., Ibarra I., Lujambio A., Jimenez Sabinina V., Senturk S., Miller M., Korimerla N., Huang J. (2021). An epigenetic switch regulates the ontogeny of AXL-positive/EGFR-TKi-resistant cells by modulating miR-335 expression. eLife.

[90] Mu L., Zhao H., Yang Y., Song R. (2021). Long noncoding RNA NEAT1 aggravates sorafenib-resistance in non-small cell lung cancer via regulating miRNA-335/c-Met. J. BUON Off. J. Balk. Union Oncol..

[91] Liu J., Yao L., Zhang M., Jiang J., Yang M., Wang Y. (2019). Downregulation of LncRNA-XIST inhibited development of non-small cell lung cancer by activating miR-335/SOD2/ROS signal pathway mediated pyroptotic cell death. Aging.

[92] Liu J., Bian T., Feng J., Qian L., Zhang J., Jiang D., Zhang Q., Li X., Liu Y., Shi J. (2018). miR-335 inhibited cell proliferation of lung cancer cells by target Tra2β. Cancer Sci..

[93] Du W., Tang H., Lei Z., Zhu J., Zeng Y., Liu Z., Huang J.A. (2019). miR-335-5p inhibits TGF-β1-induced epithelial-mesenchymal transition in non-small cell lung cancer via ROCK1. Respir. Res..

[94] Gao F.Y., Liu Q.Y., Yuan L., Xuan S.Y. (2015). Upregulation of microRNA-132 in gastric cancer promotes cell proliferation via retinoblastoma 1 targeting. Mol. Med. Rep..

[95] Zhang H., Liu A., Feng X., Tian L., Bo W., Wang H., Hu Y. (2019). MiR-132 promotes the proliferation, invasion and migration of human pancreatic carcinoma by inhibition of the tumor suppressor gene PTEN. Prog. Biophys. Mol. Biol..

[96] Khalil S., Fabbri E., Santangelo A., Bezzerri V., Cantu C., Di Gennaro G., Finotti A., Ghimenton C., Eccher A., Dechecchi M. (2016). miRNA array screening reveals cooperative MGMT-regulation between miR-181d-5p and miR-409-3p in glioblastoma. Oncotarget.

[97] Chen Z., Liu S., Tian L., Wu M., Ai F., Tang W., Zhao L., Ding J., Zhang L., Tang A. (2015). miR-124 and miR-506 inhibit colorectal cancer progression by targeting DNMT3B and DNMT1. Oncotarget.

[98] Zedan A.H., Hansen T.F., Assenholt J., Pleckaitis M., Madsen J.S., Osther P.J.S. (2018). microRNA expression in tumour tissue and plasma in patients with newly diagnosed metastatic prostate cancer. Tumour. Biol..

[99] Zhang Y., Li H., Han J., Zhang Y. (2015). Down-regulation of microRNA-124 is correlated with tumor metastasis and poor prognosis in patients with lung cancer. Int. J. Clin. Exp. Pathol..

[100] Romano G., Nigita G., Calore F., Saviana M., Le P., Croce C.M., Acunzo M., Nana-Sinkam P. (2020). MiR-124a Regulates Extracellular Vesicle Release by Targeting GTPase Rabs in Lung Cancer. Front. Oncol..

[101] Ji H., Sang M., Liu F., Ai N., Geng C. (2019). miR-124 regulates EMT based on ZEB2 target to inhibit invasion and metastasis in triple-negative breast cancer. Pathol. Res. Pract..

[102] Tang W., Lu Q., Zhu J., Zheng X., Fang N., Ji S., Lu F. (2022). Identification of a Prognostic Signature Composed of GPI, IL22RA1, CCT6A and SPOCK1 for Lung Adenocarcinoma Based on Bioinformatic Analysis of lncRNA-Mediated ceRNA Network and Sample Validation. Front. Oncol..

[103] Gao Y., Yu M., Ma M., Zhuang Y., Qiu X., Zhao Q., Dai J., Cai H., Yan X. (2019). SPOCK1 contributes to the third-generation EGFR tyrosine kinase inhibitors resistance in lung cancer. J. Cell. Biochem..

[104] Miao L., Wang Y., Xia H., Yao C., Cai H., Song Y. (2013). SPOCK1 is a novel transforming growth factor-beta target gene that regulates lung cancer cell epithelial-mesenchymal transition. Biochem. Biophys. Res. Commun..

[105] Hu R.H., Zhang Z.T., Wei H.X., Ning L., Ai J.S., Li W.H., Zhang H., Wang S.Q. (2020). LncRNA ST7-AS1, by regulating miR-181b-5p/KPNA4 axis, promotes the malignancy of lung adenocarcinoma. Cancer Cell Int..

[106] Wang Y., Zhao W., Zhang S. (2020). STAT3-induced upregulation of circCCDC66 facilitates the progression of non-small cell lung cancer by targeting miR-33a-5p/KPNA4 axis. Biomed. Pharmacother..

[107] Li X., Yu Z., Li Y., Liu S., Gao C., Hou X., Yao R., Cui L. (2015). The tumor suppressor miR-124 inhibits cell proliferation by targeting STAT3 and functions as a prognostic marker for postoperative NSCLC patients. Int. J. Oncol..

[108] Jinushi T., Shibayama Y., Kinoshita I., Oizumi S., Jinushi M., Aota T., Takahashi T., Horita S., Dosaka-Akita H., Iseki K. (2014). Low expression levels of microRNA-124-5p correlated with poor prognosis in colorectal cancer via targeting of SMC4. Cancer Med..

[109] Gholami M., Zoughi M., Larijani B., Abdollahzadeh R., Taslimi R., Rahmani Z., Kazemeini A., Behboo R., Razi F., Bastami M. (2022). The role of inflammatory miRNA-mRNA interactions in PBMCs of colorectal cancer and obesity patients. Immun. Inflamm. Dis..

[110] Zahra K., Shabbir M., Badshah Y., Trembley J.H., Badar Z., Khan K., Afsar T., Almajwal A., Alruwaili N.W., Razak S. (2022). Determining KLF14 tertiary structure and diagnostic significance in brain cancer progression. Sci. Rep..

[111] Cong C., Wang W., Tian J., Gao T., Zheng W., Zhou C. (2018). Identification of serum miR-124 as a biomarker for diagnosis and prognosis in osteosarcoma. Cancer Biomark. Sect. A Dis. Markers.

[112] Zhang S., Guo L.J., Zhang G., Wang L.L., Hao S., Gao B., Jiang Y., Tian W.G., Cao X.E., Luo D.L. (2016). Roles of microRNA-124a and microRNA-30d in breast cancer patients with type 2 diabetes mellitus. Tumour. Biol..

[113] Sticht C., De La Torre C., Parveen A., Gretz N. (2018). miRWalk: An online resource for prediction of microRNA binding sites. PLoS ONE.

[114] Gyorffy B., Surowiak P., Budczies J., Lanczky A. (2013). Online survival analysis software to assess the prognostic value of biomarkers using transcriptomic data in non-small-cell lung cancer. PLoS ONE.

[115] Lanczky A., Gyorffy B. (2021). Web-Based Survival Analysis Tool Tailored for Medical Research (KMplot): Development and Implementation. J. Med. Internet Res..

[116] Li Q., Birkbak N.J., Gyorffy B., Szallasi Z., Eklund A.C. (2011). Jetset: Selecting the optimal microarray probe set to represent a gene. BMC Bioinform..

[117] Cerami E., Gao J., Dogrusoz U., Gross B.E., Sumer S.O., Aksoy B.A., Jacobsen A., Byrne C.J., Heuer M.L., Larsson E. (2012). The cBio cancer genomics portal: An open platform for exploring multidimensional cancer genomics data. Cancer Discov..

[118] Gao J., Aksoy B.A., Dogrusoz U., Dresdner G., Gross B., Sumer S.O., Sun Y., Jacobsen A., Sinha R., Larsson E. (2013). Integrative analysis of complex cancer genomics and clinical profiles using the cBioPortal. Sci. Signal..

