# Supplementary material for: miR-124 as a Liquid Biopsy Prognostic Biomarker in Small Extracellular Vesicles from NSCLC Patients
Source: Int J Mol Sci. 2023 Jul 14;24(14):11464. doi: 10.3390/ijms241411464 (PMC10380700; doi:10.3390/ijms241411464)
Supplement: Supplementary file 1 [file ijms-24-11464-s001.zip › Sanchez_miR-124_Supplementary_IJMS.pdf]

Supplementary Materials and Methods for

miR-124 as a liquid biopsy prognostic biomarker in small extracellular vesicles from NSCLC patients

Sample collection and ethical aspects.

Tissue and plasma samples were obtained from 88 NSCLC patients from the Thoracic Surgery and the Medical Oncology Services of the University Hospital La Paz, subdivided into early-stages (one exploratory cohort of 16 patients and one validation cohort of 21 patients) and advanced-stages patients (one validation cohort of 51 patients). All patients selected did not harbor driver mutations. All samples were processed following the standard operating procedures with the appropriate approval of the Human Research Ethics Committees, including informed consent within the context of research (HULP: PI-2109).

A cohort of 16 early stage I and II patients were part of a prospective non-interventional observational study followed for 5 years (60 months) by computed tomography or until relapse. These patients underwent oncological safe radical tumor resection. . We have termed this group of patients the Exploratory Cohort. The low frequency of early-stage NSCLC patients diagnosed at the initial stages has significantly limited the number of available samples for enrollment in this cohort. Furthermore, patients undergoing surgery present with small, localized tissues, often measuring less than 3cms and the pathologist requires a sufficient amount of tissue for molecular diagnosis. Despite these limitations, we collected paired fresh frozen tumor (T) and non-tumor tissues (NT), obtained at the time of surgery, as well as prospective blood serum (CIR), collected between the date of surgery and the first oncology consultation. These are valuable samples at this stage of the illness. In addition, we carefully selected patients who exhibited clinically homogeneous characteristics that could represent the overall population of early-stage NSCLC patients, as confirmed by the clinicians and medical oncologists involved in this study (Dr. de Castro and Dr. Sanchez-Cabrero). For further validation we included a validation cohort consisting of 21 additional plasma samples from early-stage I and II patients with surgical intervention that were collected following the same times and procedures.

Subsequently, the results found in early-stages cohorts were validated in a cohort of 51 samples of advanced-stages III and IV patients, non-operable. Patients in this second Validation Cohort were followed-up for 34 months (2.8 years) by computed tomography from the start of the specific cancer treatment. In this case, blood samples were collected in an EDTA tube before

starting systemic treatment. Clinicopathological data were recorded by an independent observer and were anonymized for statistical analysis.

miRNAs extraction.

At the time of tissue collection, the tissue was immediately frozen and stored at -80ºC. Total RNA, including miRNAs, from tumor and non-tumor tissue was extracted by the guanidine thiocyanate method using TRIZOL reagent (Invitrogen, CA) and purified with the miRNeasy Mini Kit (Quiagen, CA) combined with DNase treatment to ensure that only RNA molecules were present. The concentration and integrity of the RNA was quantified by NanoDrop ND-1000 (Thermofisher Scientific, EEUU).

Blood samples were processed within the first 30 minutes after collection using Vacutainer EDTA blood collection tubes. Unbroken cells and debris were removed from the blood plasma samples by two centrifugations at 3470 rpm 10' and 14000 rpm 20' at 4°C and then stored at –80C until use. Free circulating miRNAs in serum were extracted following instructions of the QIAamp Circulating Nucleic Acid protocol from 1 ml of sample volume. RNA concentration and integrity was quantified by NanoDrop ND-1000 (Thermofisher Scientific, EEUU). sEVs-miRNAs from blood plasma were extracted by exoRNeasy Serum/Plasma Midi Kit (Qiagen, Germany), including a 0.22 µm filtration step and following product directions.

qRT-PCR.

The nonspecific retrotanscription of all miRNAs from each sample was conducted using the TaqMan™ Advanced miRNA cDNA Synthesis Kit (Thermo Fisher Scientific, USA), according to the manufacturer instructions. Briefly, the Advanced miRNA cDNA synthesis Kit includes 4 reactions to ensure the amplification of all microRNAs from the same sample: poly(A) tailing reaction, Adaptor ligation reaction, Reverse transcription (RT) reaction and miR-Amp reaction.

- *Poly(A) tailing reaction*: 5ng of each sample was diluted in 2µL of RNase free water, and mixed with 0.5 µL of 10X Poly(A) Buffer, 0.5 µL of ATP, 0.3 µL of Poly(A) Enzyme and 1.7 0.5 µL of RNase-free water to a final volume of 3 µL. Samples were then placed into a thermal cycler for 45 minutes at 37C and 10 minutes at 65C.
- *Adaptor ligation reaction*: 3 µL of Poly(A) tailing reaction was mixed with 3 µL of 5X DNA Ligase Buffer, 4.5 µL of 50% PEG 8000, 0.6 µL of 25X Ligation Adaptor, 1.5 µL of RNA Ligase, 0.4 µL of RNase-free water and ligated in a thermal cycler for 60 minutes at 16C.

- *Reverse transcription (RT) reaction*: 15 µL of Adaptor ligation reaction was mixed with 6 µL of 5X RT Buffer, 1.2 µL of dNTP Mix (25 mM each), 1.5 µL 20X Universal RT Primer, 3 µL 10X RT Enzyme Mix, 3.3 µL of RNase-free water and reversed transcribed in a thermal cycler for 15 minutes at 42C and 5 minutes at 85C.
- *miR-Amp reaction*: 5 µL of RT reaction was mixed with 25 µL of 2X miR-Amp Master Mix, 2.5 µL of 20X miR-Amp Primer Mix and 17.5 µL of RNase-free water, and amplified in a thermal cycler as follows: 5 minutes at 95C, 14 cycles of 3 seconds at 95C and 30 seconds at 60C, followed by 10 minutes at 99C.

Quantitative analysis of each specific miRNA was performed using 5 µL of a dilution 1:10 of the miR-Amp reaction, 10 µL of TaqMan® Fast Advanced Master Mix (2X), and 1 µL the next TaqMan Advanced miRNA assay for each candidate: hsa-miR-7-5p, MIMAT0000252; hsa-miR-132-3p, MIMAT0000426; hsa-miR-335-5p, MIMAT0000765; hsa-miR-148a-3p, MIMAT0000243; hsa-miR10a-5p, MIMAT0000253; hsa-miR-124-3p, MIMAT0000422 and hsa-mir-9-5p, MIMAT0000441. Samples were analyzed in triplicate using the HT7900 Real-Time PCR System (Applied Biosystems, EEUU) and the settings used were 10 min at 95C and 40 cycles of 15 s at 95C followed by 1 min at 60C. The relative expression of each miRNA was calculated by $2^{(-\Delta Ct)}$ method based in comparative threshold cycles by the RQ Manager software (Thermofisher Scientific, EEUU) and calculating the value of ΔCt by subtracting the Ct values of miR-25 (recommended as endogenous control for serum free and tissue miRNAs analysis by the provider), or miR 151a for sEVs origin (as identified by our group through bioinformatics analysis, https://doi.org/10.21203/rs.3.rs-2731367/v1), from the values of each miRNA. Non template controls qPCR were performed in every plate analyzed.

Identification of microRNA potential target genes.

Bioinformatic predictive analytics of miR-124 gene targets. Predictions of the interaction algorithms for miRNAs and their 3'UTR regions were made by interrogating miRWalk v2(1), a repository of 12 miRNA-target programs (miRWalk, miRanda, miRDB, MicroT4, miRMAP, miRNAMap, miRBridge, PITA, PICTAR2, RNAhybrid, RNA22 and TargetScan) containing both predicted and experimentally validated associations. Genes for which the binding prediction was positive in at least 8 of these 12 algorithms were then selected.

RNA-Sequencing. The quality and the quantity of the total RNA extracted from the cisplatin resistant and cisplatin sensitive human NSCLC cell lines H23S and H23R has been determined in Bioanalyzer 2100 and Qubit 3.0. Poly(A)+ mRNA fraction was isolated from total RNA and cDNA libraries were

obtained following Illumina´s recommendations. Briefly, poly(A)+ RNA was isolated on poly-T oligo-attached magnetic beads and chemically fragmented prior to reverse transcription and cDNA generation. The cDNA fragments then go through an end repair process, the addition of a single 'A' base to the 3' end and then ligation of the adapters. Finally, the products are purified and enriched with PCR to create the indexed final double stranded cDNA library. The quality of the libraries was analyzed in 4200 TapeStation, High Sensitivity assay and the quantity of the libraries was determined by real-time PCR in LightCycler 480 (Roche). The cDNA libraries were sequenced by paired-end sequencing (100 x 2) on 1 lane in Illumina HiSeq 2500 sequencer. The reads obtained were mapped to the hg19 reference genome with STAR and the counts were performed with RSEM. For the differential expression we used edgeR comparing H23R and H23S conditions. Differentially upregulated genes in resistant cells were selected based on FDR ≤ 0.05 and Log2 FC ≥ 0.

In silico databases. Kaplan-Meier Plotter(2,3): We obtained the Kaplan-Meier plots of the candidates from the bioinformatics analysis for 1925 lung cancer gene expression samples. KMplot tool works with gene chip expression data of GEO, EGA and TCGA(3). The probe with best JetSet score(4) was chosen for each gene and an autoselector was used to select the best threshold in the data range as a cut-off point. TCGA (The Cancer Genome Atlas) data(5,6): We obtained the Kaplan-Meier plots of the candidates from the bioinformatics analysis for 1027 NSCLC tumors from the TCGA. We used RNAseq V2 RSEM expression data and selected the mean of the expression data for each gene as the cut-off point between groups. Only genes with significant clinical involvement in progression-free survival (PFS) and overall survival (OS) were selected in both databases. A p-value (Log-Rank test) < 0.05 was considered significant in all survival analyses.

Statistical analysis.

Qualitative data are described as absolute frequencies and percentages and quantitative data are described as mean ± standard deviation or median and quartiles. The association between qualitative variables was analyzed using the chi-square test or square test or Fisher´s exact test. For comparisons between continuous variables, the Spearman Correlation test was used. Comparisons between categorical and continuous variables were made using the Mann-Whitney U test and Wilcoxon signed-rank test. Survival analysis was performed using Kaplan-Meier analysis, comparing survival functions by group using log-rank tests. We used the mean expression as a cut-off point to subdivide the groups between high and low miR-124 or miR-132 levels. The risk associated with the variables of interest was analyzed using Cox regression. All statistical tests were

considered bilateral and p-values of less than 0.05 were considered significant. The data were analyzed using SAS 9.3 statistical software (SAS Institute, Carly, NC, USA).

Supplementary Methods References

1 Sticht, C., De La Torre, C., Parveen, A. & Gretz, N. miRWalk: An online resource for prediction of microRNA binding sites. *PLoS One* 2018; 13, e0206239, doi:10.1371/journal.pone.0206239.

2 Gyorffy, B., Surowiak, P., Budczies, J. & Lanczky, A. Online survival analysis software to assess the prognostic value of biomarkers using transcriptomic data in non-small-cell lung cancer. *PLoS One* 2013; 8, e82241, doi:10.1371/journal.pone.0082241.

3 Lanczky, A. & Gyorffy, B. Web-Based Survival Analysis Tool Tailored for Medical Research (KMplot): Development and Implementation. *J Med Internet Res* 2021; 23, e27633, doi:10.2196/27633.

4 Li, Q., Birkbak, N. J., Gyorffy, B., Szallasi, Z. & Eklund, A. C. Jetset: selecting the optimal microarray probe set to represent a gene. *BMC Bioinformatics* 2011; 12, 474, doi:10.1186/1471-2105-12-474.

5 Cerami, E. *et al.* The cBio cancer genomics portal: an open platform for exploring multidimensional cancer genomics data. *Cancer Discov* 2012; 2, 401-404, doi:10.1158/2159-8290.CD-12-0095.

6 Gao, J. *et al.* Integrative analysis of complex cancer genomics and clinical profiles using the cBioPortal. *Sci Signal* 2013; 6, pl1, doi:10.1126/scisignal.2004088.
