# Supplementary material for: miR-124 as a Liquid Biopsy Prognostic Biomarker in Small Extracellular Vesicles from NSCLC Patients
Source: Int J Mol Sci. 2023 Jul 14;24(14):11464. doi: 10.3390/ijms241411464 (PMC10380700; doi:10.3390/ijms241411464)
Supplement: Supplementary file 1 [file ijms-24-11464-s001.zip › SuppFig1.pdf]

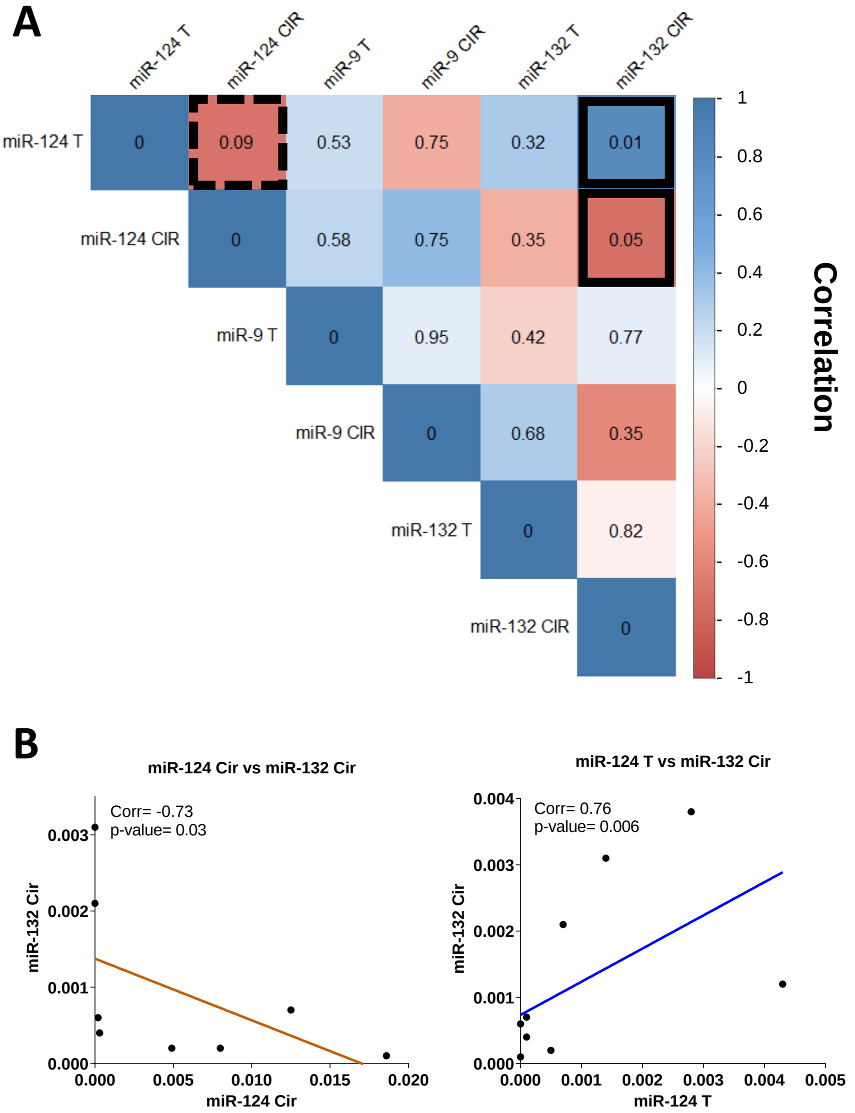

Supplementary Figure S1. Correlation analysis between expression level tissue (intracellular) and circulating miRNAs. A) *Spearman correlation test comparing different miRNAs contrast. The color scale represents the correlation, and the significant p-value are indicated by black box.* ***B)*** *Only significant correlation analysis from A) are represented. In blue, significant positive relationship and in red, significant negative relationship. P-value < 0.05 were considered significant. Corr:* [illegible]
